# Salinity Moderated the Toxicity of Zinc Oxide Nanoparticles (ZnO NPs) towards the Early Development of *Takifugu obscurus*

**DOI:** 10.3390/ijerph20043209

**Published:** 2023-02-12

**Authors:** Yuqing Lin, Jun Wang, Huichao Dai, Feijian Mao, Qiuwen Chen, Hanlu Yan, Mo Chen

**Affiliations:** 1 Center for Eco-Environmental Research, Nanjing Hydraulic Research Institute, Nanjing 210029, China; 2 State Key Laboratory of Hydrology-Water Resources and Hydraulic Engineering, Nanjing Hydraulic Research Institute, Nanjing 210029, China; 3 Yangtze Institute for Conservation and Green Development, Nanjing 210029, China; 4 China Three Gorges Corporation, Wuhan 430010, China

**Keywords:** *Takifugu obscurus*, ZnO nanoparticles, salinity, oxidative stress, survival

## Abstract

ZnO nanoparticles (ZnO NPs) have been applied in a wide range of fields due to their unique properties. However, their ecotoxicological threats are reorganized after being discharged. Their toxic effect on anadromous fish could be complicated due to the salinity fluctuations during migration between freshwater and brackish water. In this study, the combined impact of ZnO NPs and salinity on the early development of a typical anadromous fish, obscure puffer (*Takifugu obscurus*), was evaluated by (i) observation of the nanoparticle characterization in salt solution; (ii) quantification of the toxicity to embryos, newly hatched larvae, and larvae; and (iii) toxicological analysis using biomarkers. It is indicated that with increased salinity level in brackish water (10 ppt), the toxicity of ZnO NPs decreased due to reduced dissolved Zn^2+^ content, leading to higher hatch rate of embryos and survival rate of larvae than in freshwater (0 ppt). The irregular antioxidant enzyme activity changes are attributed to the toxic effects of nanoparticles on CAT (catalase), but further determination is required. The results of present study have the significance to guide the wildlife conservation of *Takifugu obscurus* population.

## 1. Introduction

Nanoparticles (NPs) are commonly referred to particles with at least one dimension less than 100 nm [1]. The small sizes can lead to several unique physiochemical properties, and NPs are enabled to achieve outstanding optical sensitivity, reactivity, and conductivity compared with the bulk materials [2]. According to Bouwmeester et al. [3], with the revolutionary development of nanotechnology, various types of NPs are increasingly used in numerous commercial and industrial products. Among different types of NPs, the metal oxide nanoparticles (MNPs) have been mass produced for over a decade and applied in consumer goods, agriculture, and industry [4]. In particular, zinc oxide nanoparticles (ZnO NPs) are considered to be one of the most widely employed, which have shown the capabilities of photocatalysis and photooxidation [5]. As mentioned by Keller, McFerran, Lazareva, and Suh [6], the annual global production of ZnO NPs is supposed to be over 30,000 metric tons, and the final disposals are landfills, soils, water bodies, or the atmosphere, which could be habitats for massive organisms. Therefore, the potential ecotoxicological risks of ZnO NPs has recently become a hot topic.

ZnO NPs released from various sources such as transport, industry, and agriculture appear to eventually accumulate in aquatic systems. The contaminated water bodies could lead to a large amount of interactions with the aquatic ecosystems [7]. Previous studies have shown the toxicity of ZnO NPs to different kinds of aquatic organisms including vertebrates, invertebrates, algae, plants, and bacteria [8,9,10]. The main attention of these prior toxicity studies has been paid to fish, especially the zebrafish, because of its comprehensively understood biological mechanism as one of the model test organisms approved by regulatory agencies [11]. For example, the experiment conducted by Bai et al. [12] based on zebrafish embryos 96 h after fertilization (hpf) showed that exposure to ZnO NPs (30 nm) could cause the effects of killing embryos, retarding hatching, reducing larval length, and tail deformities. In addition, ZnO NPs can accumulate to toxic concentrations in mammals through aquatic and terrestrial food chains [13]. As summarized by Golbamaki et al. [14], ZnO NPs could be cytotoxic and genotoxic to human epidermal, liver, and kidney cells, as significant cell and DNA damages were observed post exposure in several previous research. Regarding the possible toxicity mechanisms, Zhu et al. [15] suggested the combined influence of nanoparticle aggregates and dissolved Zn^2+^, while Yu et al. [16] proposed that aggregation and precipitation could contribute to the reduction of toxicity. According to Bacchetta et al. [17], the soluble Zn^2+^ passing through the cell membrane and combining with mitochondria can activate the apoptotic pathway to promote the production of reactive oxygen species (ROS) and trigger the autophagy process upon oxidative stress. Thus, ZnO NPs undoubtedly pose great potential hazards to aquatic organisms and the ecosystem and are thus regarded to be “extremely toxic” [18].

The current ZnO NPs hazard assessments are commonly based on traditional toxic effect tests under one designed condition, which can lead to biased results for overlooking the differences of the environment in each region and time period [17]. There are types of aquatic ecosystems worldwide, including freshwater, marine, lake, river, pond, and wetlands, with distinct characteristics of temperature, salinity, and pH [19]. Hence, it should be noted that research on the toxic effects of ZnO NPs under the impact of different environmental factors could be important for further understanding and assessing their ecotoxicological risks [9]. According to Wagner et al. [20], ZnO NPs can be less toxic under pH 6.5 to pH 8.5 since their control of nanoparticles’ surface charge could decrease the solubility, thereby facilitating aggregation and lowering stability. Additionally, the study using *Thalassiosira pseudonana* carried out by Yung et al. [21]. indicated that ZnO NPs at higher temperature and salinity could constitute aggregates in larger volume and produce Zn^2+^ in lower concentration, resulting in lowered toxicity. The experiment conducted by Lai et al. [22] obtained a similar result that high salinity could reduce the toxic effects of ZnO NPs to the copepod *Tigriopus japonicus*, which is possibly because of the improved osmotic pressure of copepods and ion dissolution with lower salinity. Based on relevant experimental results, raising salinity is considered to be a potential moderator against the toxicity of metal oxide nanoparticles [23]. Therefore, the influences of salinity on the physical and chemical properties of ZnO NPs could have a great research value.

In order to explore the combined toxic effects of salinity and ZnO NPs in different aquatic ecosystems, the optimal sample to ensure the accuracy of the experimental results is anadromous migratory fish due to its strong ability adapting to salinity changes during migration processes [24]. As a typical anadromous fish mainly distributed in the Chinese coastal waters and the middle and lower Yangtze River, juveniles of obscure puffer (*Takifugu obscurus*) move from rivers and lakes to offshore seas after being hatched and fatten, indicating large fluctuations in salinity levels for the juveniles [23,25]. The environmental stress during their vulnerable early development periods could cause a negative impact on the growth, migration, and reproduction processes of individuals or the population [26]. Hence, it is necessary to understand the capacity and response of obscure puffer embryos and larvae to the nanoparticles’ potential toxicity under the influence of salinity. As for the specific defense mechanism against the oxidative stress caused by ZnO NPs, the research of Kim et al. [27] on the resistance of *T. obscurus* to cadmium toxicity confirmed the function of highly expressed antioxidant genes. The produced harmful superoxide anion could be converted into H_2_O_2_ through superoxide dismutase (SOD) and further decomposed into H_2_O and O_2_ through catalase (CAT) [28,29]. According to Frankič et al. [30], malondialdehyde (MDA) is one of the ultimate products of lipids peroxidation and has been evaluated in dozens of related studies due to its reflection ability of cellular damages through toxins. Furthermore, the adenosine triphosphatases (ATPases) index is another important biochemical parameter for obscure puffer juveniles because of their undeveloped intestinal osmoregulation system and dominated Na^+^/K^+^ ion-transport regulation [31]. Therefore, the determination of those four biochemical indicators could contribute to the further identification of the toxicity mechanism of nanoparticles on the obscure puffer juveniles.

To evaluate the potential impacts of ZnO NPs and salinity on the early development of *T. obscurus*, this work aimed to quantify the toxic effects via the hatching rate of embryos and the survival rate of larvae as well as determine the toxicity mechanism based on the configuration of nanoparticles and the measurement of biochemical indices including CAT, SOD, MDA, and Na^+^/K^+^ ATPase.

## 2. Materials and Methods

All experiments in this study were approved by the ACUC (Animal Care and Use Committee) of Nanjing Normal University, Nanjing, China, and carried out according to its guidelines (Research Permit Number: SYXK2015-0028).

### 2.1. Preparation of Embryos and Larvae

After collection from a fish farm located at Yangzhong City, Jiangsu Province, China, the *T. obscurus* adults were conveyed to lab. The adult fish were temporarily stored in a fish tank (100 L freshwater), whose environmental circumstance were similar to the fish farm. Based on the method of Yang and Chen [25], the luteinizing-hormone-releasing hormone analogue was applied on 3 males and 1 female of adult fish, and fertilized eggs were acquired. The eggs were then retained in a tank with temperature of 23 ± 1 °C, pH value of 7.1 ± 0.3, and constant aeration. Damaged and dead fertilized eggs was removed after 16 h via wide-mouth pipettes. Part of the healthy eggs were transported for subsequent embryo-related experiments. The eggs were slowly stirred with feathers to evenly dispense the eggs. The remaining eggs were used for the hatching process for collection of larvae. Larvae in good health were randomly selected for further experiments around 24 h after hatching.

### 2.2. Preparation of ZnO NPs

The ZnO NPs (diameter = 50 nm) were purchased from Nanjing Haitai Nanoparticles Ltd. The stock solution of ZnO NPs (0.5 g/L) was prepared before the exposure treatments. Nanoparticles with a diameter around 25 nm were suspended in Milli-Q water, which was further processed in an ice-water bath for half an hour using the ultrasonic vibration at 40 kHz. The concentration of the stock ZnO NPs solution was 0.5 g/L, which was then diluted to six levels, including 0 mg/L, 5 mg/L, 10 mg/L, 20 mg/L, 50 mg/L, and 100 mg/L. In order to assess the morphological configuration of ZnO NPs, H-7650 scanning electron microscopy (SEM, Hitachi, H-7650, Tokyo, Japan) was employed for the suspension.

### 2.3. Experimental Procedure

Based on a full factorial experiment with three replicates for each treatment and two elements, the salinity was employed as 0 ppt and 10 ppt, and the concentration of ZnO NPs was employed as 0 mg/L, 5 mg/L, 10 mg/L, 20 mg/L, 50 mg/L, and 100 mg/L. Tissue culture-treated (TCT) 6-well plates containing 3 mL of ZnO NPs solution in different concentrations were applied to rear 10 embryos or larvae per well. The body length of newly hatched larvae is generally less than 1 mm, suggesting that the capacity of 16.7 mL per well is adequate. For the adjustment process of embryos or larvae to the salinity changes, 2 ppt was raised per hour with continuous aeration for each solution under the salinity of 10 ppt. The replacement of half ZnO NPs solution with newly prepared solution was then carried out daily to ensure no significant alteration of the salinity and nanoparticles levels. The culture conditions were set for all tests with temperature at 23 ± 1 °C, dissolved oxygen content over 5.2 mg/L, pH value adjusted to 7.1 ± 0.3, and duration of light and dark to 12 h each. For each embryos-based treatment, the hatching rate of embryos and the survival rate of new larvae 24 h post hatching were recorded. For each larvae-based treatment, the dead larvae were removed, and the survival rate was recorded every 24 h for 96 h.

### 2.4. Determination of Biochemical Parameters

In order to determine the damage extent and toxicity mechanism of ZnO NPs to *T. obscurus* larvae, the larvae-based treatments with 50 mg/L ZnO NPs (the concentration approximately corresponds to lethal dose 50%) under 0 ppt and 10 ppt of salinity condition were selected for the measurement of biomarkers. Ten experimental larvae were respectively and randomly selected at 0, 24, 48, 72, and 96 h. According to Sun et al. [32], the biomarkers of hatched larvae with relatively less body length could be determined through homogenates of whole fish. Prior to the homogenization process in a Dounce homogenizer, larvae were rinsed using 0.7% normal saline with a temperature around 0 °C and dried through filter papers. After that, the larvae were each transferred in 2 mL of 0.7% normal saline. The centrifugation (4000× *g*) procedure at 4 °C was then performed for 10 min for the elimination of homogenate’s fragments of cartilages and cells, and the supernatants were used for the measurement of biomarkers. In this study, the diagnostic reagent kits for the determination of CAT (U/mg protein), SOD (U/mg protein), MDA (nmol/mg protein), and Na^+^/K^+^ ATPase (μmol Pi/mg protein/h) were purchased from the NanJing Jian Cheng Bioengineering Institute (China). The diameters of ZnO NPs at 0.5, 1, 6, 12, and 24 h were detected by Dynamic Light Scattering (DLS, Nicomp, Z3000, Tokyo, Japan).

### 2.5. Statistical Analysis

Two levels of salinity were selected in the exposure experiment―0 ppt represents freshwater, and 10 ppt represents brackish water. Four days of exposure was determined due to no living larvae observed after 96 h of cultivation with 100 mg/L ZnO NPs under 0 ppt. Differences between treatments are calculated by one-way ANOVA and Tukey’s multiple comparison. Each result was displayed as mean ± standard error (SE), and *p* < 0.05 was considered as statistically significant. All data analysis and graphs were conducted with SigmaPlot 11.0.

## 3. Results

### 3.1. Characterization of ZnO NPs in Salinity Solution

**Figure 1:**
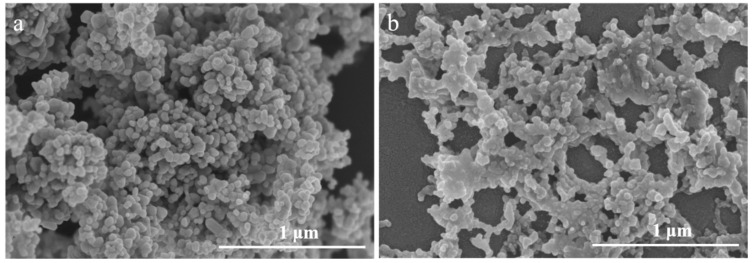
SEM images of ZnO NPs suspension (50 mg/L) under 10 ppt (**a**) and 0 ppt (**b**).

As shown in Figure 1, nanoparticles aggregated into diameter of about several hundred nanometers in various shapes and sizes. Brackish water could lead to more aggregates with relatively larger dimensions and less dissolution than freshwater (Figure 1).

### 3.2. Toxic Effects of ZnO NPs on Embryos

**Figure 2:**
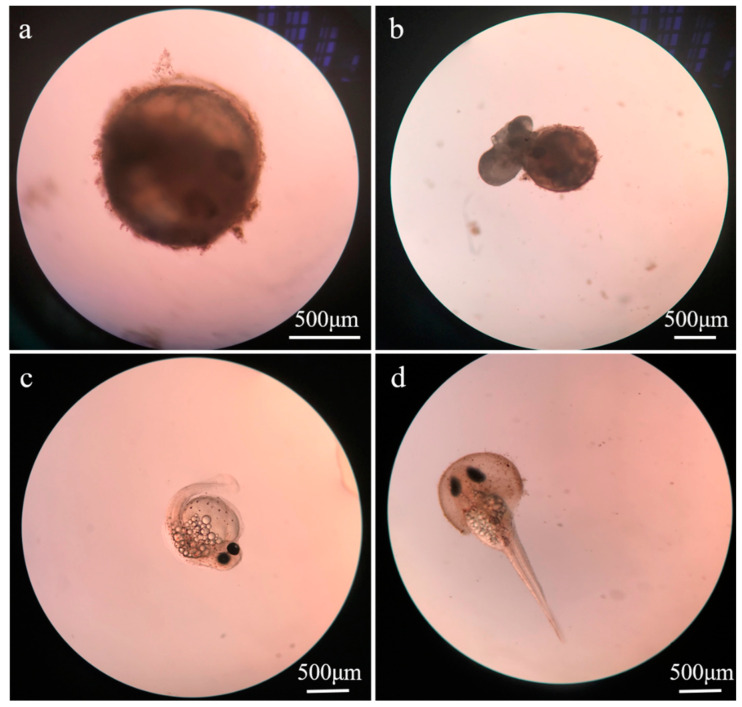
Effects of ZnO NPs (50 mg/L) on embryos and larvae. (**a**) The surface of embryo was covered with ZnO NPs; (**b**) the yolk sac of embryo was overflowed; (**c**) abnormal larvae with caudal vertebra bending; (**d**) the larvae could not completely hatch out.

**Figure 3:**
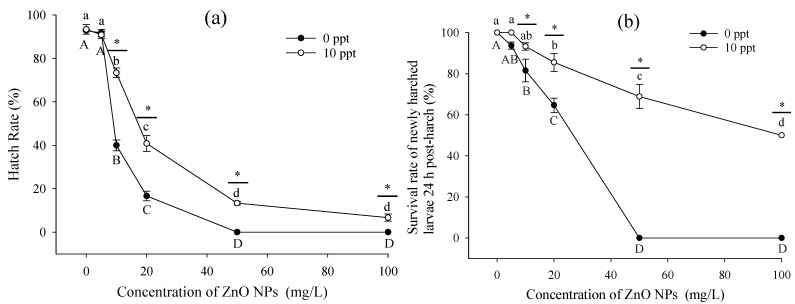
(**a**) Hatch rate of *T. obscurus* embryos under different ZnO NPs concentrations and salinities and (**b**) survival rate of *T. obscurus* larvae 24 h post hatch under different ZnO NP concentrations and salinities. Different letters represent significant differences (*p* < 0.05) between different ZnO NPs concentrations under the same salinity. The asterisks above the black lines represent significant differences (*p* < 0.05) between different salinities under the same concentration of ZnO NPs (*n* = 3).

ZnO NPs fully covered the surface of the embryo (blastocystic egg of approximately 24 h), resulting in the overflow of embryo yolk sac (Figure 2). As a result, the hatching of larvae (healthy young fish surviving within 24 h after hatching out) was affected, and the larvae had deformities. The toxicity to *T. obscurus* embryos was evaluated based on the hatching rate of eggs and the survival rates of newly hatched larvae. According to Figure 3a, the hatching rate of embryos decreased significantly with increasing ZnO NPs concentration and the decrease of salinity. The hatch rate was 0 with 50 mg/L ZnO NPs in freshwater, while it was around 13.3 ± 0.8% with the same nanoparticles concentration in brackish water. Higher ZnO NPs concentrations and lower salinity level could lead to lower survival rate of larvae (Figure 3b), which was consistent with the results of hatch rate. Under 0 ppt, no larvae were hatched with over 50 mg/L ZnO NPs; thus, the survival rate was regarded to be 0. In contrast, the survival rates in saline water were higher than 50%.

### 3.3. Toxic Effects on Larvae

**Figure 4:**
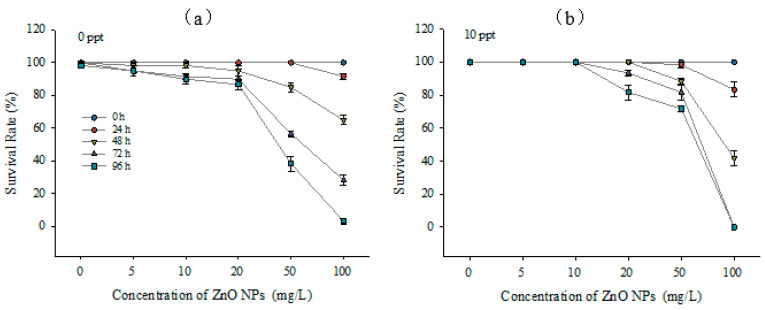
Survival rate of *T. obscurus* larvae under different salinities (**a**) 0 ppt, and (**b**) 10 ppt, exposure time, and ZnO NPs concentrations (*n* = 3).

The survival rates of *T. obscurus* larvae after exposing to a range of ZnO NPs concentrations for different time periods in freshwater and brackish water are displayed in Figure 4. Generally, the survival rate decreased significantly with higher ZnO NPs concentrations and longer exposure time. The survival rate of each treatment was higher than 80% after 24 h of exposure, while it decreased to 0 with 100 mg/L ZnO NPs after 96 h of exposure. As for the impacts of salinity levels on the toxicity, it can be observed that the death of *T. obscurus* larvae was observed with over 10 mg/L ZnO NPs under 10 ppt. In contrast, death was observed without the presence of nanoparticles under 0 ppt. No survival was observed after cultured for 96 h under both salinity conditions with 100 mg/L solution. Similarly, survival was hardly observed for the treatment in brackish water with the same concentration already after 72 h.

**Figure 5:**
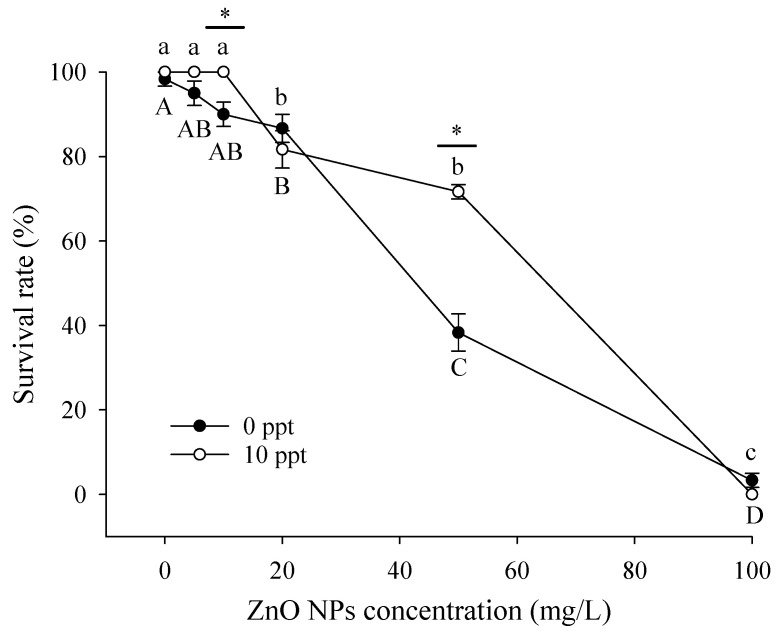
Survival rate of *T. obscurus* larvae under different ZnO NP concentrations and salinity levels after 96 h of exposure. Different letters represent significant differences (*p* < 0.05) between different ZnO NPs concentrations under the same salinity. The asterisks above the black lines represent significant differences (*p* < 0.05) between different salinities under the same concentration of ZnO NPs (*n* = 3).

In order to determine the influence of salinity, Figure 5 shows the survival rate of *T. obscurus* larvae after 96 h of exposure to different ZnO NP concentrations and salinity. The survival rates of higher salinity condition were significantly higher than that under 0 ppt with ZnO NPs concentrations of 0, 5, 10, and 50 mg/L. It should be noted that the surviving larvae had varying extents of diseases, including pericardial effusion and edema.

### 3.4. Biochemical Responses of Larvae

**Figure 6:**
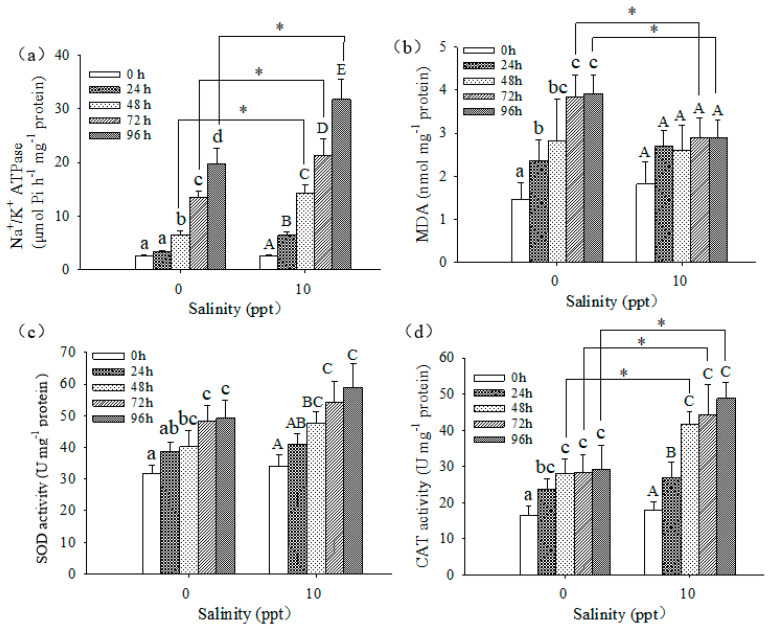
Na^+^/K^+^ ATPase (**a**), MDA (**b**), SOD (**c**), and CAT (**d**) in the whole *T. obscurus* larvae with exposure to 50 mg/L ZnO NPs for 96 h under two salinities (0 ppt and 10 ppt). Different letters represent significant differences (*p* < 0.05) between different exposure times under the same salinity. The asterisks above the black lines represent significant differences (*p* < 0.05) between different salinities under the same ZnO NPs concentration and exposure time (*n* = 3).

Figure 6 shows the Na^+^/K^+^ ATPase values under exposure to 50 mg/L of ZnO NPs with different salinity levels. Na^+^/K^+^ ATPase values increased significantly with the increase of salinity level and exposed time. Similar to Na^+^/K^+^ ATPase, CAT activity also showed significant higher values in brackish water than freshwater after 48 h of exposure. Another antioxidant indicator, SOD activity also had higher values with 10 ppt of salinity at each duration time although not statistically significant. In contrast, MDA showed a different pattern, with no significant differences for various exposure time in brackish water and significantly higher values under 0 ppt than 10 ppt after 72 h.

## 4. Discussion

### 4.1. Impacts of Salinity on the Characterization and Toxicity of ZnO NPs

ZnO NPs aggregated in higher extent in brackish water than freshwater (Figure 1), indicating the change of characterization of ZnO NPs in solution by salinity. Similar results are demonstrated in several examples of previous research. For example, Yung et al. [33] concluded that increased salinity could lead to greater diameters and lower concentration of dissolved Zn^2+^. According to Kim et al. [34], aggregation could be achieved through counteracting with the particle entropy and electrostatic energy barrier. However, higher salinity in brackish water can eliminate most of the particle electrostatic repulsion in the solution [35], which results in larger aggregates. The salt could provide extra chloride ions for the complexation reaction with the dissolved metal ion and reduce the concentration of Zn^2+^ [36,37]. Therefore, with increased salinity, the amount of aggregation increases, and the content of Zn^2+^ decreases.

Many studies showed that the dissolved metal ions are responsible for their negative impacts on aquatic organisms, which means that the relatively lower concentration of dissolved Zn^2+^ in brackish water could lead to the lower toxic effects of ZnO NPs than in freshwater [9,38,39]. The experiment conducted by Adam et al. [40] tested the chronic toxic effects of ZnO NPs and dissolved Zn^2+^ to *Daphnia magna* separately for 21 days and emphasized that the ZnO NPs’ toxicity is highly likely due to the dissolution instead of the aggregations or nanoparticles. The toxicological effects experiments on *T. obscurus* in this study showed similar results, as the survival rates of larvae were lower under 0 ppt than 10 ppt with 10 and 50 mg/L ZnO NPs (Figure 4), indicating the main contribution of zinc ions to the toxicity.

### 4.2. Embryos and Newly Hatched Larvae

As the most vulnerable stage in the life cycle of *T. obscurus*, the embryo could be easily affected by various environmental stresses, including the combined effects of salinity and ZnO NPs [41]. In the present study, the concentration of zinc oxide nanoparticles was positive correlated with the mortality of embryo (Figure 3). However, the research of Browning et al. [42] showed that the toxic effects of gold nanoparticles on zebrafish embryos was not related with their concentration, and about 74% of the embryos managed to develop into juveniles without deformation. The difference could be due to the distinct physiochemical properties of different nanoparticles [43]. Thus, further selection of nanomaterials regarding toxicity is needed before applications [44].

Hua et al. [45] explored the toxicity of different shapes of ZnO NPs to zebrafish embryos and suggested that nanoparticles in each suspension could be more toxic than dissolved Zn^2+^. According to Zhao et al. [46], Zn^2+^ dissolution promotes the toxic effects of ZnO NPs to some extent, while pure nanoparticles could cause oxidative stress, DNA damages, and developmental toxicity on embryos. Therefore, in this study, the significant decrease of embryos’ hatching rate and of survival rate of the newly hatched larvae can be mainly attributed to the toxicity of nanoparticles.

### 4.3. Biochemical Responses of Larvae

As a membrane protein, Na^+^/K^+^ ATPase promotes the active exchange between K^+^ ions and Na^+^ ions, provides energy for ion transport, and maintains the osmotic pressure across the membranes [47]. In our study, the Na^+^/K^+^ ATPase activity in brackish water was significantly higher than in freshwater for each treatment with 50 mg/L ZnO NPs, indicating its adaptive changes to salinity. Na^+^/K^+^ ATPase activity also increased significantly with exposure time at the same salinity level (Figure 6). Similar results were reported in several previous studies. For example, the experiment using rainbow trout carried out by Richards [48] showed that Na^+^/K^+^ ATPase α1b mRNA (one of the isoforms) increased rapidly after transferred to 80% seawater and continued to rise with time. Kong et al. [49] also showed a similar combined effect of exposure time, temperature, and salinity on the Na^+^/K^+^ ATPase activities of mud crab muscles. Hence, in the present study, salinity and ZnO NP had joint toxicity to the survival of *T. obscurus* embryos and larvae.

The effects of nanoparticles can stimulate or inhibit the activities of various antioxidant enzymes [17,50,51]. As the first defense line, SOD converts superoxide anion O^2-^ into H_2_O_2_ and oxygen, and the generated H_2_O_2_ is further converted into H_2_O and oxygen by CAT, minimizing the adverse effects of ROS [51]. One previous study demonstrated that ZnO and CuO nanoparticles in *Cumumis sativus* could lead to alterations of oxidative stress, resulting in statistically significant increased SOD and CAT activities [52]. In the present study, SOD and CAT had higher activities in brackish water than freshwater (Figure 5). Under brackish condition, the activities of both SOD and CAT increased significantly with time, indicating antioxidative properties, whereas CAT activity remained constant and SOD activity increased under freshwater condition. Similar results have been demonstrated in previous studies. The inhibition of CAT activity under the influence of ZnO NPs can lead to the incomplete removal of H_2_O_2_, which can result in the accumulation of ROS in cells [46,53]. The changes of peroxidase activity could indicate that the ROS stress is greater for *T. obscurus* larvae in freshwater, which can be further confirmed with MDA content. MDA is one of the products of the reaction in cell membrane based on unsaturated fatty acids and free radicals [46]. It is generally regarded as an indicator of the oxidative stress and the extent of cell destruction under stress [54]. The experiment conducted by Xiong et al. [55] showed the increased MDA content with increased TiO_2_ NPs concentration in zebrafish. In this study, MDA content was significantly higher in freshwater than brackish water (Figure 5) and consistent with the results of hatch rate and survival rate of embryos and newly hatched larvae (Figure 3). These results indicated that embryos were under less stress in brackish water than in freshwater. Therefore, it is proposed that the toxic effects of ZnO NPs to *T. obscurus* larvae could hinder the activities of antioxidant enzymes (mostly CAT) and cause abnormal function, which could be the reason for the high mortality of embryos and larvae with the concentration of 50 mg/L ZnO NPs.

## 5. Conclusions

In conclusion, this study evaluated the impact of ZnO NPs on the early development of *T. obscurus* based on the quantification and analysis of their toxic effects on embryos and larvae. The toxicity is associated with exposure time and nanoparticle concentration. Salinity negatively affected the toxicity of ZnO NPs. The influence of increased salinity on ZnO NPs was mainly manifested in the formation of aggregations with greater size and less content of dissolved Zn^2+^. Thus, salinity is an important factor to be considered when evaluating the impact of nanoparticles on aquatic organisms.

## Data Availability

Data is available based on proper request to the corresponding author.
